# Plateau of practice effects and noise with repeat SDMT testing in multiple sclerosis

**DOI:** 10.1177/13524585251344794

**Published:** 2025-05-31

**Authors:** Lenka Novakova, Igal Rosenstein, Markus Axelsson, Menno M Schoonheim, Ralph HB Benedict, Tom A Fuchs

**Affiliations:** Department of Clinical Neuroscience, Institute of Neuroscience and Physiology, Sahlgrenska Academy, University of Gothenburg, Gothenburg, Sweden; Region Västra Götaland, Department of Neurology, Sahlgrenska University Hospital, Gothenburg, Sweden; Department of Clinical Neuroscience, Institute of Neuroscience and Physiology, Sahlgrenska Academy, University of Gothenburg, Gothenburg, Sweden; Region Västra Götaland, Department of Neurology, Sahlgrenska University Hospital, Gothenburg, Sweden; Department of Clinical Neuroscience, Institute of Neuroscience and Physiology, Sahlgrenska Academy, University of Gothenburg, Gothenburg, Sweden; Region Västra Götaland, Department of Neurology, Sahlgrenska University Hospital, Gothenburg, Sweden; MS Center Amsterdam, Department of Anatomy and Neurosciences, Amsterdam Neuroscience, Amsterdam University Medical Center, Location VUmc, Vrije Universiteit Amsterdam, Amsterdam, The Netherlands; Jacobs Multiple Sclerosis Center, Department of Neurology, Jacobs School of Medicine and Biomedical Sciences, University at Buffalo, The State University of New York, Buffalo, NY, USA; MS Center Amsterdam, Department of Anatomy and Neurosciences, Amsterdam Neuroscience, Amsterdam University Medical Center, Location VUmc, Vrije Universiteit Amsterdam, Amsterdam, The Netherlands

**Keywords:** Multiple sclerosis, cognition, cognitive decline, Symbol Digit Modalities Test, practice plateau, remote testing

## Abstract

**Background:**

The Symbol Digit Modalities Test (SDMT) is the most widely used test of cognition in people with multiple sclerosis (PwMS), and repeated testing is confounded by test–retest noise and practice effects.

**Objective:**

To investigate the extent to which SDMT practice effects build and plateau with high-frequency testing, reliable cutoffs for longitudinal change, and whether short-interval testing improves detection of cognitive decline.

**Methods:**

PwMS were tested with the SDMT monthly across 3 years. Plateau regression analyses were used to determine inflection points of practice effects, and the reliable-change was assessed. To evaluate effects of testing density on cognitive decline detection, this sample was compared with a separate low-density testing group.

**Results:**

The study included 71 people with relapsing-remitting MS (77.5% female), mean (standard deviation (SD)) age 37.3 (9.3), with 27.8 (21.3) SDMT assessments over 3.2 (2.4) years. The plateau of practice effects was reached after 18 repetitions (*p* < 0.001). Within this sample, ⩾7-point SDMT change was needed to detect cognitive decline with 90% confidence. Higher testing density did not improve detection of cognitive decline (*p* = 0.256).

**Conclusion:**

We observed practice effects building for eighteen SDMT assessments and test–retest variability consistent with literature. These results provide guidance on SDMT which should be accounted for with alternate versions and reliable-change methodologies.

## Introduction

The Symbol Digit Modalities Test (SDMT)^1^ is the most widely used test of cognition in people with multiple sclerosis (PwMS). It is a reliable and valid test in clinical settings^2,3^ and is used as an endpoint in clinical trials.^4^ Recently, it was shown that improvements in SDMT performance are more pronounced when using the same SDMT form repeatedly, especially at shorter intervals.^5^ It is likely that practice effects reach a plateau after sufficient repetitions, although this has not yet been investigated.

In clinical trials and in clinical practice, distinguishing between practice effects and true treatment effects is essential for accurately assessing therapeutic efficacy.^4^ It is often assumed that practice effects eventually stabilize, and any subsequent increase in performance reflects genuine clinical improvement, although this has not been tested. In addition, with the growth of remote cognitive testing, practice effects associated with repetitive SDMT testing are likely to become saturated.^6–11^ It is therefore important to understand how practice effects build and plateau with repeated assessments, how much change exceeds noise inherent to the test and whether high-density testing improves detection of cognitive decline.

We therefore had the following objectives:

1. Characterize (A) the degree to which practice effects build with repeated SDMT administrations and (B) where these effects plateau.
2. Evaluate reliable-change intervals for capturing longitudinal SDMT decline.
3. Determine whether short-interval testing improves detection of cognitive decline.

This study used a pre-existing retrospective data set, where participants were administered the same version of the SDMT. In future analyses, prospective data with more strictly structured SDMT assessments, as well as alternate SDMT forms, are preferable.

## Materials and methods

Patient written consent was provided for patient inclusion in the Swedish MS registry. The Swedish Ethics Review Authority (No. 2023-03410-02) approved this study, and the research board of Swedish MS registry approved the data withdrawal. Anonymized data used in this article will be shared on reasonable request from any qualified investigator for questions related to the published article.

### Study cohort

The clinical data on PwMS were extracted from the Swedish MS registry,^12^ a nationwide web-based platform collecting data prospectively. We extracted data on two separate patient cohorts:

1. The high-density testing group, in which patients treated with natalizumab were tested with the SDMT monthly as a screening instrument for progressive multifocal leukoencephalopathy between 2007 and 2015. The patients started the monthly testing during this period the following treatment initiation with natalizumab. The same SDMT version was used each time. This high-density SDMT repetition group is a unique data set – with up to 90 SDMT repetitions per patient and testing occurring at brief intervals (~30 days). This makes this data set valuable for studying practice effects of the SDMT and their effect on clinical interpretation of longitudinal change.
2. The low-density testing group, in which SDMT was administered every 6 months between 2010 and 2021 as part of clinical routine, used alternating SDMT versions. At baseline, these patients had following treatment: natalizumab (72.2%, *n* = 319), fingolimod (9.7%, *n* = 43), dimethyl fumarate (6.3%, *n* = 28), no treatment (3.6%, *n* = 16), rituximab (2.3%, *n* = 10), alemtuzumab (2%, *n* = 9), teriflunomide (1.8%, *n* = 8), interferon beta (1.1%, *n* = 5), glatiramer acetate (0.7%, *n* = 3) and unknown (0.2%, *n* = 1) (see Table 1 for details).

**Table 1.** Sample Characteristics.

	High frequency	Low frequency	*p*
Number of subjects	71	380	
Age, mean years (SD)	37.3 (9.3)	35.6 (10.1)	0.151
Sex (female), no (%)	55 (77.5%)	269 (77.5%)	0.235
Disease duration, mean years (SD)	7.4 (7.4)	6.5 (6.4)	0.354
Baseline EDSS, median (IQR)	2 (1.5-3)	2 (1.5-3)	0.233
Follow-up time, mean years (SD)	3.2 (2.5)	6.8 (2.8)	**<0.001**
Baseline SDMT, mean (SD)	53.4 (13.4)	55.6 (15)	0.216
Days between SDMTs, mean (SD)	32.7 (4.9)	341.2 (153.6)	**<0.001**

EDSS = Expanded Disability Status Scale, IQR = interquartile range, SD = standard deviation, SDMT = Symbol Digit Modalities Test.

The patient data were extracted from the registry for this study if they met the following criteria: relapsing-remitting MS diagnosis, >18 years old, no other neurologic disorder, and performed SDMT between 2007 and 2021. Only oral versions of SDMT were used. Patients were excluded if they had ⩽2 SDMT assessments. Subjects from the low-density group were also excluded if they performed SDMT more often than twice a year.

### Cognitive testing

Oral forms of the SDMT were administered by trained nurses. The standard SDMT version^1^ was used for the high-frequency testing. In alternate SDMT versions, used for the low-density testing cohort, the same symbols were presented as in the standard SDMT version. However, there was a new arrangement of the pairings between symbol and number, that is, the key was different.^13^ Thus, alternate forms were comparable with the standard version, but the key was changed randomly by trial coordinators.

### Statistical analysis

#### Aim 1

Characterize (A) the degree to which practice effects build with repeated SDMT administrations and (B) where these effects plateau.

First, to evaluate the degree to which practice affects build with repetitive SDMT assessments and eventually plateau, we applied plateau linear regression analyses. The linear plateau model fits a segmented regression model that follows two phases: a positive linear response and a flat plateau. This statistical model is used to describe data where the continuous response variable increases linearly with the independent variable up to a certain threshold (plateau), after which it remains constant.^14^ Thus, we applied this methodology to establish an estimated slope, describing the relationship between SDMT repetition number and SDMT score performance (e.g. improved performance with repeated test administration). Then, with this methodology, we also established a group-level inflection point, after which a ‘plateau’ occurs, where the positive relationship between SDMT repetition and SDMT performance is no longer observed. This was then repeated on an individual-subject level. In addition, because the practice plateau may be different for high- and low-density SDMT testing, we applied this analysis method separately for the high- and low-density SDMT repetition groups.

The retrospective data sets used for plateau analysis were limited. Therefore, to estimate the distribution and variability of the linear plateau model parameters, we performed a non-parametric bootstrap procedure. Specifically, we resampled the data set with replacement 1,000 times and fit a linear plateau model in each iteration. From the resulting bootstrap distribution of the estimated change point, we computed empirical quantiles at 5% intervals (from the 5th to the 95th percentile) to summarize the uncertainty and variability in the estimated threshold.

#### Aim 2. Determine reliable-change intervals for capturing longitudinal SDMT decline

Previous studies have applied the reliable-change-index (RCI) to identify statistically reliable cutoffs for detecting longitudinal change on the SDMT, ones that exceed expected test–retest noise.^15,16^ These studies have indicated an 8-point change as a reliable cutoff for capturing longitudinal SDMT change in people with MS. However, these studies calculated RCI using wide time intervals (6-12 months) and with only one testing repetition.^16^ We therefore aimed to validate these findings using a shorter test–retest interval and across multiple test repetitions. To do this, we applied the RCI as described by Weinstock et al.^15^ Here, we used a two-assessment sliding window across the first 15 repetitions in the high-density testing data set to calculate the test–retest variability and RCI cutoffs (80% and 90% confidence) across these time-points. We choose 15 repetitions because more than 70% of samples had ⩾15 repetitions.

#### Aim 3. Determine whether short-interval testing improves detection of cognitive decline

Finally, to evaluate whether high-density SDMT testing improves the likelihood of observing cognitive decline, we performed a logistic regression analysis predicting confirmed (sustained over 3 months) reliable-change (⩾8 points) cognitive decline^15,16^ (yes/no) using testing density (low vs. high) as an independent factor. To account for differences in the high-density and low-density cohorts in the regression analysis, we also included the following covariates: sex, age, disease duration, follow-up time and therapy efficacy.

All statistical analyses were performed in R version 4.3.2 and IBM SPSS version 28.

## Results

### Sample description

The characteristics of the sample are presented in Table 1. The high-density testing group included 71 PwMS, with a mean (standard deviation (SD)) age of 37.3 (9.3) years; 77.5% were female. Mean (SD) disease duration was 7.4 (7.4) years, and the mean follow-up time in this study was 3.2 (2.5) years. There were 2599 unique SDMTs, the mean baseline SDMT was 53.4 (13.4) points, the mean number of SDMTs per person was 27.8 (21.3) and the mean time between SDMTs was 32.7 (4.9) days. 92% of patients had at least 5 SDMT assessments, 83% had at least 10 SDMT assessments, 75% had at least 15 SDMT assessments, 62% had at least 20 SDMT assessments, 55% had at least 25 SDMT assessments and 52% had at least 30 SDMT assessments. During follow-up, 9 patients had relapse, 7 patients radiological disease activity and 2 patients had relapse plus radiological disease activity.

The low-density testing group included 380 PwMS, with a mean (SD) age of 35.6 (10.1) years; 77.5% were female. Mean (SD) disease duration was 6.5 (6.4) years, and a mean follow-up time in this cohort was 6.8 (2.8) years. There were 3415 unique SDMTs; the mean baseline SDMT was 55.6 (15.0) points, the mean number of SDMTs per person was 5.8 (7.2) and the mean time between SDMTs was 341.2 (153.6) days.

### Aim 1. Characterize (A) the degree to which practice effects build with repeated SDMT administrations and (B) where these effects plateau

**Figure 1.**
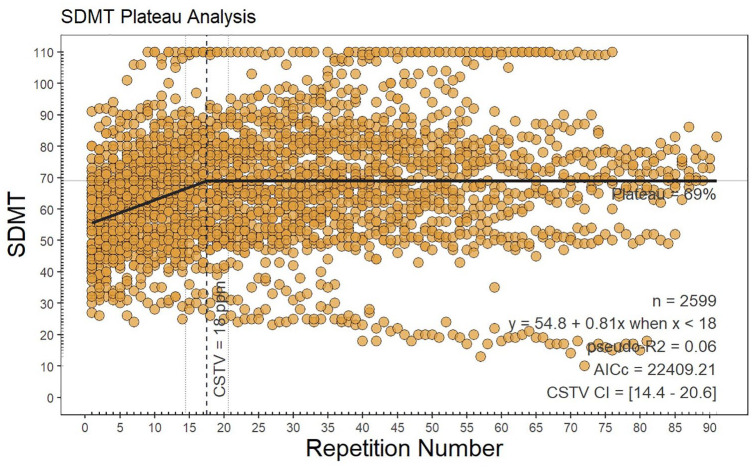
Plateau analysis. This plateau analysis plot shows the relationship between the Symbol Digit Modalities Test (SDMT) scores and the number of SDMT repetitions. Each dot represents an individual SDMT score for a specific repetition number. A total of 2599 data points are plotted. A black line indicates the trend in SDMT scores as the repetition number increases. The trend line shows an initial positive slope, indicating an increase in SDMT scores with repetition, that is, practice effects. Change in slope at the 18th repetition is indicated by a vertical solid line representing plateau of practice effects. The trend indicates that SDMT scores generally improve with repetition up to the 18th repetition, after which the scores show levelling out of practice effects. AICc: corrected Akaike Information Criterion, CI: confidence interval, CSTV: Critical Soil Test Value, SDMT: Symbol Digit Modalities Test.

Using the high-density SDMT testing cohort, practice effects persisted, and a group-level plateau of practice effects was reached after 18 repetitions (95% confidence interval = 14.4–20.6, *p* < 0.001). Prior to the plateau, SDMT scores improved at a mean rate of 0.54 points per repetition, shown in Figure 1, finally arriving at a plateau mean SDMT score of 69 points.

The subject samples were insufficient to identify plateaus at an individual-subject level. However, following bootstrapping (1000 times with replacement), the linear plateau at the 50th percentile occurred at 21.1 SDMT repetitions, with the 25th–75th percentile range being 11.0–35.0. All values at 5% intervals are provided in the Supplementary Material.

The same analysis excluding patients with a ceiling effect, that is, reaching the maximal number of 110 points in SDMT, and patients with progressive decline, that is, having 30 or less points in SDMT at baseline, was performed, and the plateau was reached after 24 repetitions (95% CI = 20.9–27.3, *p* < 0.001; Supplementary Material, Figure 1S). The same analysis excluding patients with relapse (*n* = 11) is shown in Supplementary Material, Figure 2S.

There was no SDMT plateau reached on the set of PwMS with the low-density SDMT testing. Therefore, no plateau analysis could be performed. Instead, we have illustrated a non-linear model fit (Supplementary Material, Figure 3S). Using a linear mixed-effects model, the estimated increase per SDMT repetition was 0.14 points (*p* = 0.007) and including the effect of time, we found 1.4 points decline per year (*p* < 0.001).

### Aim 2. Determine reliable-change intervals for capturing longitudinal SDMT decline

**Figure 2.**
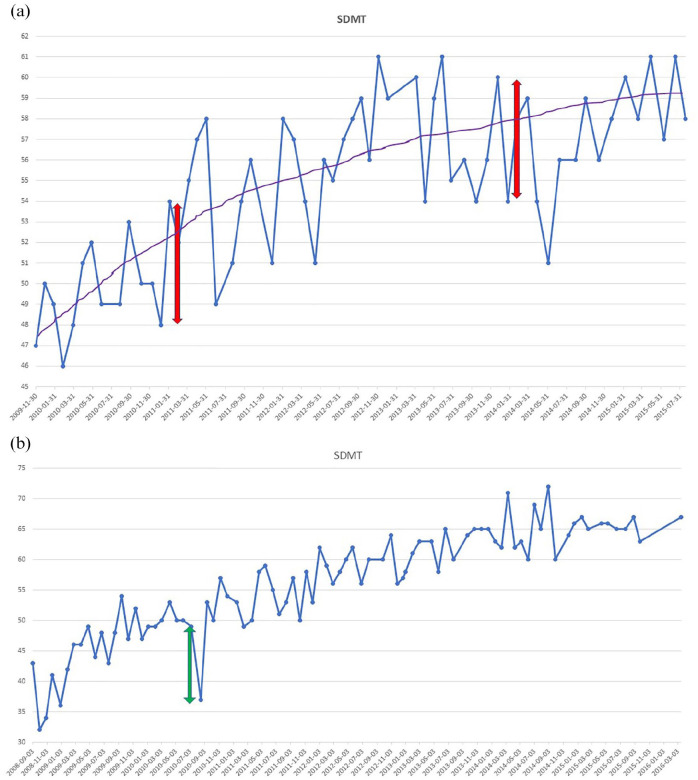
Repeated Symbol Digit Modalities Test assessments of an individual patient. The line graph shows the change of Symbol Digit Modalities Test (SDMT) scores over a series of dates for a representative patient. The blue dots represent the SDMT scores for a specific date, and the blue line connecting them shows how the SDMT scores fluctuate, indicating variability in the scores for each test date. (a) The purple line represents the overall trend in the SDMT scores, indicating an improvement in scores over time. The red double-headed arrows highlight specific periods where there is a 6-point change in SDMT that is unlikely to represent statistically meaningful change, given expected variance for this test. (b) A green double-headed arrow indicates a possible cognitive relapse with a negative change of 12 points followed by a positive change of 16 points.

In the high-density data set, using a sliding window of two consecutive assessments across 15 SDMT repetitions, the mean(SD) RCI cutoff was ± 6.39 (1.89) at an 80% CI and ± 8.18 (2.34) at a 90% CI. In Figure 2(a), we present an SDMT of a representative individual patient from the high-density SDMT testing cohort that exemplifies how frequent testing captures natural fluctuations (test–retest variability) in SDMT scores rather than clinical cognitive decline. Figure 2(b) is an example of a patient that may have experienced a cognitive relapse^17^ with recovery and slowly increasing SDMT performance due to practice effects. Due to the recovery of SDMT performance, this example does not represent ‘confirmed’ cognitive worsening.

### Aim 3. Determine whether short-interval testing improves detection of cognitive decline

To evaluate whether high-density SDMT testing improves the likelihood of observing cognitive decline, we performed a logistic regression predicting confirmed (sustained over 3 months) reliable-change cognitive decline using testing density as an independent factor. The previously published reliable-change cutoff of ⩾8 points was used to prevent issues from using the same data set for RCI determination and use.^15^

Total time of observation was significantly longer for the low-density testing group (*t* = −11.014, *p* < 0.001) (see Table 1 for details). Results for this analysis are presented in Table 2. The percentage of confirmed significant SDMT decline was 43.4% in the low-density and 12.9% in the high-density group. The probability of confirmed SDMT decline was not influenced by the density of testing [odds ratio (OR) = 1.66 (95% CI = 0.67–3.98), *p* = 0.256]. However, longer follow-up time was associated with an increasing likelihood of detecting significant SDMT decline [OR = 1.5 (95% CI = 1.36-1.65), *p* < 0.001]. Other covariates (age, sex, disease duration and choice of the therapy) did not significantly impact the probability of observing SDMT decline.

**Table 2.** Logistic regression model predicting likelihood of detecting significant confirmed SDMT decline.

	Variable	Coefficient	Odds ratio	CI lower	CI upper	*p*
Independent	Density of testing (low)	0.507	1.660	0.692	3.979	0.256
Covariates	Age	-0.025	0.975	0.950	1.001	0.055
	Sex	-0.100	0.905	0.551	1.487	0.694
	Disease duration	-0.003	0.997	0.959	1.038	0.900
	Follow-up time	0.405	1.500	1.360	1.655	**<0.001**
	Therapy efficacy (low)	-0.432	0.649	0.377	1.119	0.120
	Therapy efficacy (none)	0.268	1.307	0.424	4.031	0.641

## Discussion

In this study, including a unique patient data set with monthly SDMT assessments, we observed that practice effects with increasing SDMT scores build with repeated SDMT assessments, only reaching a plateau after 18 repetitions. We also showed that individual patient test–retest variability observably occurs over 0–8 points between tests. Finally, high-density SDMT testing did not increase the likelihood of detecting cognitive decline within individual subjects, whereas longer total follow-up did.

### Plateauing of practice effects

Persistence of practice effects with eventual plateau has not been investigated previously. However, in a similar study including patients treated with natalizumab, repeated monthly SDMTs continuously improved in PwMS for 26-month follow-up, with 1.2 points per test during the first 6 months and by 0.4 points per test thereafter,^18^ which is on average 0.6 point per test during the follow-up, similar to the results with 0.54 point increase per repetition in our cohort. Interestingly, a new key reverted the SDMT score to baseline after 2 years, supporting the use of alternating testing forms to reduce practice effects. Our results are congruent with previous findings,^5,19,20^ especially considering that no plateau was reached in the low-density cohort with the spaced repetition and form variation.

In clinical settings with testing occurring every 1–3 years, it is unlikely that patients would reach the practice plateau, as the practice effects surprisingly continue to build even after more than 10 repetitions. In clinical settings, practice effects may also be managed with spaced repetitions and alternating testing forms.^5^ Alternate forms have also been used in a larger Swedish national study showing the stable trajectories in SDMT over 7 years following the initiation of disease-modifying therapy.^13^ In the present study, there was a slight decline in SDMT and no practice effect overall in the low-density testing group. However, there is still a need for a consensus on frequency in cognitive assessment of MS patients.

Notably, our results only pertain to oral SDMT; hence, this study should be repeated with digital symbol-digit tests and other forms. In several observational and validation studies in tablet- or smartphone-based versions of SDMT were presented.^11,21–25^ Despite the adaptation of smartphone SDMT with randomly generated symbols to prevent memorization, learning still may occur, compound with SDMT itself, and might also continue to build beyond 20 test repetitions.^9^ This is in line with the results of plateau analysis in our study.

### Statistically meaningful cognitive change

Our RCI analysis and example cases (Figure 2) illustrate the natural fluctuations (test–retest variability) seen with repeated SDMT testing. The cutoffs identified (6–8 points) were consistent, albeit slightly less conservative relative to previously published RCI cutoffs (8–10).^15,26^ The usage of RCI is crucial for accurately identifying cognitive decline or improvement at the individual level,^5,15,26^ providing a threshold for what constitutes a statistically ‘reliable’ change, based on the standard error of measurement, test–retest error, and the reliability of the test. This threshold can better guide clinical decisions. However, confirmation of decline is also likely an important component for distinguishing temporary from persistent cognitive decline.^27^

### The influence of testing frequency on detection of cognitive decline

In our study, alternating forms and assessment density did not increase the likelihood of observing cognitive decline (Table 2) after controlling for baseline characteristics and total follow-up time. Although we might miss transient cognitive decline,^28^ most cognitive declines with reduction in SDMT last 1.5 years post-relapse^29^ and high-density testing with repeated forms may interfere with the measurement of subtle decline accumulating over time. The SDMT does not accurately reflect steady cognitive decline, when it was used in clinical trials every 24 weeks,^19^ it showed a steady increase during the 3-year follow-up. Especially given the timeframe of clinical trials and our result of reaching the plateau after 18 months, this finding is relevant when using SDMT as an outcome measure. Similarly, performance increased using the same form with exposure <2 years apart in clinical practice.^5^ Despite testing intervals of 6 months or longer, the practice effects are still present in clinical studies^19^ and clinical practice.^5^

### Limitations

First, our plateau determination analysis was completed on a high-density retrospective data set in which patients had variable amounts of assessments. Although the testing interval remained close to 30 days (mean = 32.7, SD = 4.9), some patients were not individually assessed more than 15 times (25% of the sample). This may partly explain why plateau analysis revealed plateau on a group level, but not for an individual-subject basis. The bootstrapping analysis completed to address sample limitations and instability of our findings resulted in a wide range of potential plateau thresholds, ranging from 11 to 35 in the middle 50th percentile of bootstrapped samples. Thus, more research is needed to validate our findings.

The retrospective data set that we used for short-interval SDMT testing analysis was structured with repetitions of the same SDMT form, rather than recommended alternating forms. Using the short-interval testing with the same form in our study, we likely increased practice effects per repetition^5^ and it may have allowed the patients to arrive at the plateau earlier. However, it is not known how much the practice effects are delayed by using the alternative forms. A prospective study with appropriately structured data is needed to build on our findings.

In addition, because the rates of confirmed cognitive worsening in this sample were low, we cannot exclude that the plateau of learning effects interferes with the ability to capture cognitive decline. In addition, the low-density testing group was larger than the high-density group. The relatively small sample size of high-density group has affected the statistical inference, and the CI is therefore wide. Furthermore, the PwMS in high-density group were homogeneously treated, while the low-density group had both low- and high-efficacy therapies. However, the therapy choice was done in clinical practice based on the disease activity. Thus, treatment with low efficacy was considered sufficient for a particular patient, but the majority of patients were treated with high-efficacy therapy. Notably, although, there was no effect of treatment choice in the regression analysis.

## Conclusion

In conclusion, SDMT practice effects persisted up to one and a half years after 18 SDMT repetitions of the same form, and high-density testing did not improve the likelihood of capturing cognitive decline. We also showed test–retest variability consistent with the literature. If and how alternate forms can prevent learning effect and detection of cognitive decline needs to be further investigated. This variability between tests and surprising consistency of practice effects over many repetitions support the use of reliable-change methodologies and appropriately spaced cognitive assessments.

## Supplemental Material

sj-docx-1-msj-10.1177_13524585251344794 – Supplemental material for Plateau of practice effects and noise with repeat SDMT testing and in multiple sclerosis Supplemental material, sj-docx-1-msj-10.1177_13524585251344794 for Plateau of practice effects and noise with repeat SDMT testing and in multiple sclerosis by Lenka Novakova, Igal Rosenstein, Markus Axelsson, Menno M Schoonheim, Ralph HB Benedict and Tom A Fuchs in Multiple Sclerosis Journal
