# Supplementary material for: Plateau of practice effects and noise with repeat SDMT testing in multiple sclerosis
Source: Mult Scler. 2025 May 31;31(9):1121–9. doi: 10.1177/13524585251344794 (PMC12357967; doi:10.1177/13524585251344794)
Supplement: sj-docx-1-msj-10.1177_13524585251344794 – Supplemental material for Plateau of practice effects and noise with repeat SDMT testing and in multiple sclerosis [file sj-docx-1-msj-10.1177_13524585251344794.docx]

**Supplementary material**

**Figure 1S**


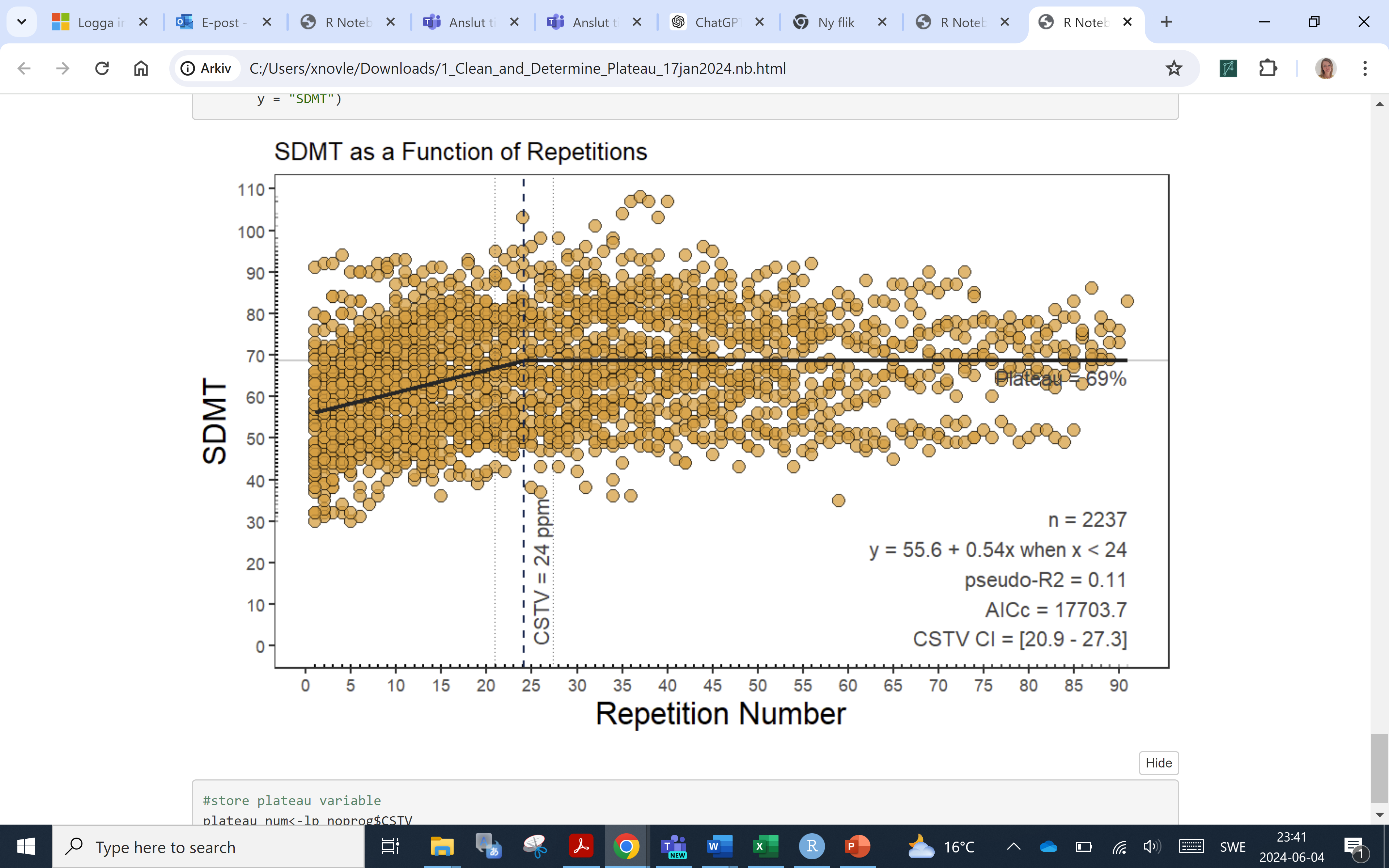


This plateau analysis plot shows the relationship between the Symbol Digit Modalities Test (SDMT) scores and the number of SDMT repetitions. Each dot represents an individual SDMT score for a specific repetition number. A total of 2237 data points are plotted. A black line indicates the trend in SDMT scores as the repetition number increases. The trend line shows an initial positive slope, indicating an increase in SDMT scores with repetition, i.e. practice effects. Change in slope at the 24th repetition is indicated by a vertical solid line representing plateau of practice effects. The trend indicates that SDMT scores generally improve with repetition up to the 24th repetition, after which the scores show leveling out of practice effects.

For this analysis, we excluded patients with ceiling effect, i.e. reaching the maximal number of 110 points in SDMT, and patients with progressive decline, i.e. having 30 or less points in SDMT at baseline with declining number of points during the follow-up (n=7) with corresponding SDMTs (n=363).

AICc=corrected Akaike Information Criterion, CI=confidence interval, CSTV= Critical Soil Test Value, SDMT=Symbol Digit Modalities Test

**Figure 2S**

**
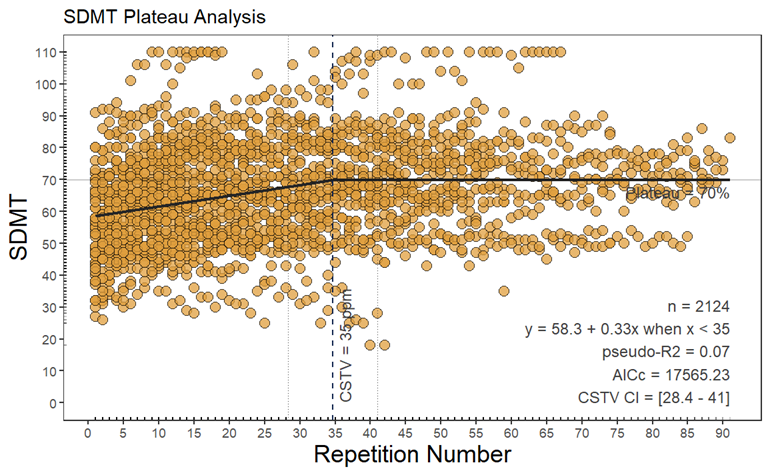
**

This plateau analysis plot shows the relationship between the Symbol Digit Modalities Test (SDMT) scores and the number of SDMT repetitions. Each dot represents an individual SDMT score for a specific repetition number. A total of 2124 data points are plotted. A black line indicates the trend in SDMT scores as the repetition number increases. The trend line shows an initial positive slope, indicating an increase in SDMT scores with repetition, i.e. practice effects. Change in slope at the 35th repetition is indicated by a vertical solid line representing plateau of practice effects. The trend indicates that SDMT scores generally improve with repetition up to the 24th repetition, after which the scores show leveling out of practice effects.

For this analysis, we excluded patients with relapse.

AICc=corrected Akaike Information Criterion, CI=confidence interval, CSTV= Critical Soil Test Value, SDMT=Symbol Digit Modalities Test

**Figure 3S.** Plot with SDMT and number of repetitions in the low-density repetition group


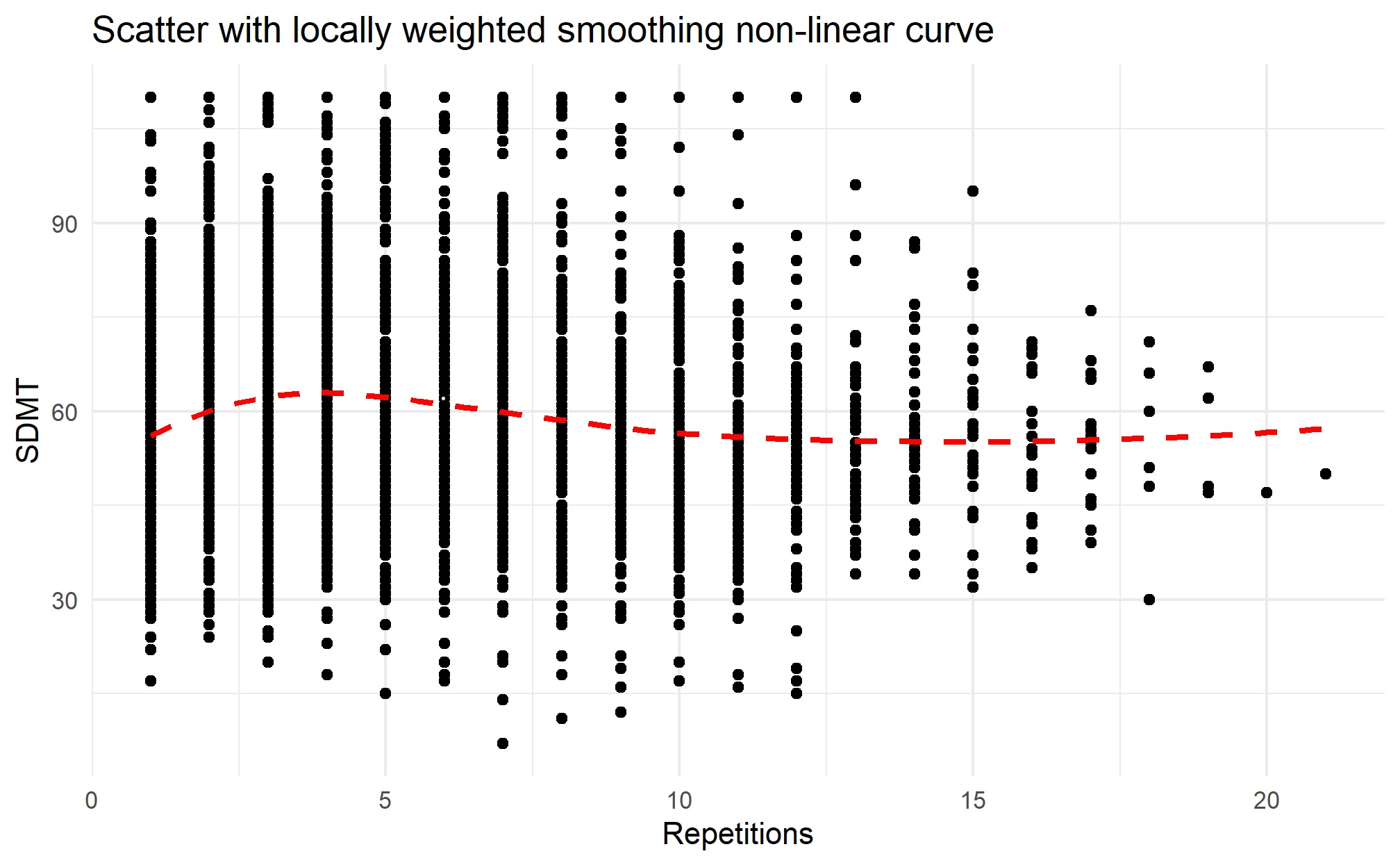


**Supplementary Table.** Bootstrapped plateaus with corresponding percentiles

| Percentile | Value |
| --- | --- |
| 5% | 5.0000038 |
| 10% | 6.6666667 |
| 15% | 8.0000028 |
| 20% | 9.3288285 |
| 25% | 11 |
| 30% | 12.600627 |
| 35% | 15.09349 |
| 40% | 16.999999 |
| 45% | 19.143773 |
| 50% | 21.155498 |
| 55% | 24.348793 |
| 60% | 27.132901 |
| 65% | 29.890001 |
| 70% | 31.985699 |
| 75% | 35.000001 |
| 80% | 40 |
| 85% | 44.49139 |
| 90% | 52.486153 |
| 95% | 65.041128 |
